# Multidrug-resistant Gram-negative bacterial infections and associated factors in a Kenyan intensive care unit: a cross-sectional study

**DOI:** 10.1186/s12941-023-00636-5

**Published:** 2023-09-14

**Authors:** Jane Wairimu Maina, Frank Gekara Onyambu, Peter Shikuku Kibet, Abednego Moki Musyoki

**Affiliations:** 1 Department of Medical Laboratory Science, The Nairobi West Hospital, Nairobi, Kenya; 2 Department of Medical Laboratory Science, Kenyatta University, Nairobi, Kenya; 3 Meru University of Science and Technology, Nairobi, Kenya

**Keywords:** Multidrug-resistance, Gram-negative bacteria, Infections, Risk factors

## Abstract

**Background:**

Patients admitted to intensive care units (ICU) are at risk of Gram-negative bacteria (GNB) infections, especially those caused by multidrug-resistant (MDR) isolates, increasing morbidity, mortality, and healthcare costs. However, epidemiological surveillance data on MDR bacteria to inform infection prevention and control (IPCs) interventions is limited in our study setting. Here we assessed the prevalence and factors associated with GNB infections in ICU- patients admitted in our study setting.

**Methods:**

This was a hospital-based cross-sectional study among patients admitted to ICU at the Nairobi West Hospital, Kenya, between January and October 2022. Altogether, we recruited 162 patients, excluding those hospitalized for less than 48 h and declining consent, and collected demographics and clinical data by case report form. Blood, wound and throat swab, ascetic tap, stool, urine, tracheal aspirate, and sputum samples were collected cultured. Isolates identity and antimicrobial susceptibility were elucidated using the BD Phoenix system.

**Results:**

The prevalence of GNB infections was 55.6%, predominated by urinary tract infections (UTIs). We recovered 13 GNB types, with *Escherichia coli* (33.3%) and *Klebsiella pneumoniae* (31.1%) as the most common isolates. Factors associated with GNB infections were a history of antibiotic use (aOR = 4.23, p = 0.001), nasogastric tube use (NGT, aOR = 3.04, p = 0.013), respiratory tract (RT, aOR = 5.3, p = 0.005) and cardiovascular (CV, aOR = 5.7, p = 0.024) conditions. 92% of the isolates were MDR,predominantly *Escherichia coli, Klebsiella pneumoniae,* and *Pseudomonas aeruginosa*.

**Conclusion:**

We report a high prevalence of MDR-GNB infections, predominated by UTI, in ICU, whereby patients with a history of antibiotic use, using the NGT, and having RT and CV conditions were at increased risk. To improve the management of ICU-admitted patients, continuous education, training, monitoring, evaluation and feedback on infection prevention and control are warranted in our study setting.

## Introduction

Gram-negative bacteria (GNB) infections, including respiratory tract, urinary tract, wound or surgical site, and bloodstream infections, are among the leading causes of morbidity, mortality, and increased healthcare costs in patients admitted to intensive care units (ICUs) [1–3]. ICU-admitted patients are more vulnerable to GNB infections because of frequent invasive medical procedures, including intubation, mechanical ventilation, and vascular access [4]. Additionally, reduced immune response due to trauma, surgery, and sepsis and impaired protective mechanisms, such as cough, swallowing reflexes, gastric acid secretion, and normal flora, predispose ICU-admitted patients to infections [5].

ICUs are often the epicenter of multidrug-resistant (MDR) GNB, mainly arising from the frequent and inappropriate or incorrect use of broad-spectrum antibiotics that drive drug-resistant strains evolution [1, 2] and bacterial exchange of resistance traits, including plasmid-encoded β-lactamases, aminoglycosides modifying enzymes, quinolone resistance gene, in the environment through horizontal gene transfer [3]. Additionally, poor adherence to infection prevention and control (IPC) policies substantially contributes to the high burden of MDR infections in ICUs [4].

MDR-GNB infections, especially those caused by extended-spectrum beta-lactamases (ESBL)- and carbapenemases-producing *Enterobacteriaceae* and non-fermenters, such as *Pseudomonas aeruginosa, Acinetobacter baumannii and Stenotrophomonas maltophilia*, are present clinically with limited therapeutic options [5] ICU-admitted patients with antibiotic-resistant bacterial infections have worse clinical outcomes than non-resistant strains and have a significant economic burden [6]. Those with cardiovascular disease, urinary catheterization and inappropriate empirical antibiotic therapy show increased mortality [7].

Even though a given organism antibiograms should guide the choice of antibiotics for MDR infection [8, 9], in resource-constrained settings, clinical laboratories are inadequately equipped and poorly supplied, and the personnel capacity is underdeveloped. For instance, in a point prevalence survey across 14 Kenyan public hospitals, only 2 (0.1%) of 1505 patients received treatment based on antibiogram, and 697 (46.4%) were inappropriately prescribed antibiotics [10] with a potential negative impact on antimicrobial resistance [11]. MDR bacterial infections pose a substantial clinical challenge in Kenya [12, 13]. In the Kenyatta National Hospital’s 2015 annual antimicrobial surveillance data, 88% of pathogens isolated were MDR, whereas 26% were extensively drug-resistant [14]. Continuous and systematic antimicrobial resistance surveillance in line with local and global AMR control action plans is warranted. Here, we determined the prevalence and factors associated with MDR-GNB infections and mortality in a Kenyan tertiary hospital ICU. This information is critical for antimicrobial therapy selection and evaluating the effectiveness of AMR infection prevention and control strategies.

## Materials and methods

### Study setting, design and population

We conducted this study at The Nairobi West Hospital (TNWH), a 400-bed capacity, including an 18-bed intensive care unit (ICU), a private tertiary healthcare facility in Nairobi City, Kenya. This was a hospital-based cross-sectional study among patients admitted to the ICU at the Nairobi West Hospital, Kenya, between January and October 2022. A total of 162 patients were recruited, excluding those hospitalized for less than 48 h and declining consent. We obtained informed consent for study participation for each patient through a close relative or a family legal representative, carried out the research project in accordance with the Declaration of Helsinki, observed participants well-being, and ensured the doctors in charge of the patients got results timely on all critical findings. The Kenyatta University Ethical Review Committee (Protocol no. PKU/2395/11531), National Commission for Science and Innovation (License No. NACOSTI/P/22/15238), and TNWH management approved the research project.

### Sample collection

We collected the participant’s demographics and clinical presentation data using a structured questionnaire and case report forms. The samples collected depended on the patient’s clinical presentation. A qualified nurse collected the tracheal aspirate and ascitic tap samples into sterile containers. Swab samples were collected using sterile swabs (Delta lab, Spain), whereas urine samples were collected aseptically from a catheter collection port using a needle into 20 mL sterile screw-capped universal containers (Delta lab, Spain). A 2-inch of catheter distal tip was clipped directly into a sterile container and transported at room temperature to microbiology laboratory within 15 min to avoid drying. For blood samples, we obtained 8–10 mL of participants' blood using a needle and syringe into BD BACTEC™ Blood Culture Media (BD Diagnostics, Sparks, MD, USA). All samples were transported to TNWH Microbiology laboratory in a cool box and processed within 2 h.

### Bacterial isolation, identification, and antimicrobial susceptibility testing

We used standard bacteriological methods for bacterial isolation [15, 16]. Briefly, urine samples were inoculated on cysteine–lactose electrolyte deficient agar (CLED) (HI Media Laboratories LLC, India) and incubated aerobically at 37 °C overnight. Pus swab, ascetic tap, sputum, and tracheal aspirate samples were inoculated on MacConkey agar (Hi Media Laboratories LLC, India), sheep blood agar (Hi Media Laboratories LLC, India), and chocolate blood agar (CBA) (Hi Media Laboratories LLC, India), and incubated at 37 °C overnight at both ambient air and 5% CO_2_. We loaded blood samples in the BD BACTEC™ Automated Blood Culture System (BD Diagnostics, Sparks, MD, USA) at 36 °C for up to 5 days, and positive-flagged samples sub-cultured on MacConkey agar (Hi Media Laboratories LLC, India), sheep blood agar (Hi Media Laboratories LLC, India), and CBA (Hi Media Laboratories LLC, India), and incubated at 37 °C overnight at both ambient air and 5% CO_2._

Isolates’ identity and antimicrobial susceptibility were elucidated using the BD Phoenix system (BD Diagnostics, Sparks, MD, USA), following the manufacturer’s instructions. Clinical and laboratory standards institute guidelines [17] informed the choice of test antibiotic and inhibition zones interpretation. The antibiotic panels were: amoxicillin/clavulanic acid (4/2–16/2 μg/ml), ampicillin (4–16 μg/ml), piperacillin/tazobactam (4/4–64/4 μg/ml), trimethoprim/sulfamethoxazole (1/19–4/76 μg/ml), nitrofurantoin (16–64 μg/ml), gentamicin (2–8 μg/ml), amikacin (8–32 μg/ml), ceftriaxone (1–32 μg/ml), cefazolin (4–16 μg/ml), cefotaxime (4–16 μg/ml), ceftolozane/tazobactam (1/4–8/4 μg/ml), ceftazidime (2–16 μg/ml), cefepime (1–16 μg/ml), tigecycline (1–4 μg/ml), ciprofloxacin (0.5–2 μg/ml), levofloxacin (1–4 μg/ml), meropenem (0.5–4 μg/ml), ertapenem (0.25–2 μg/ml), imipenem (0.25–4 μg/ml) and colistin (1–4 μg/ml). *Pseudomonas aeruginosa* (ATCC 27853) and *Escherichia coli* (25922) were used as the standard control organisms.

We defined carbapenem resistance as resistance to either ertapenem (≥ 2 μg/ml), imipenem (≥ 4 μg/ml), or meropenem (≥ 4 μg/ml), whereas resistance to either ceftriaxone (≥ 4 μg/ml) or ceftazidime (≥ 16 μg/ml) as third-generation cephalosporin resistance [21]. Isolates resistant to three or more antibiotic classes were considered multidrug-resistant (MDR) [13].

### Statistical analysis

We analyzed the data using the Statistical Package for the Social Sciences (SPSS) version 17.0 for Windows (IBM SPSS Statistics, IBM Corporation, Armonk, NY, USA). We also analyzed the data for normality and presented it in figures and tables, with categorical data in frequencies and percentages and continuous data in means and medians. We used binomial logistic regression analysis to determine the association between GNB infections, ICU admission outcomes, and patients’ socio-demographic and clinical characteristics. Any association with p-value ≤ 0.2 were further analyzed by multinomial logistic regression, with the statistical significance level set at p < 0.05 ((95% Confidence Interval (95% CI)) and statistically significant associations bolded in Table 2.

## Results

### Socio-demographic and clinical characteristics of study participants

We sampled 162 critically ill patients admitted to an intensive care unit at the Nairobi West Hospital in Kenya. The study participants constituted a diverse group, aged between 1 and 88 years, with a mean of 44.2 years and a 17.42 standard deviation. The majority were males (64.2%, 104/ 162)-adults (92.6%, 150/162)-not referrals from other healthcare facilities (69.8%, 113/162). The median length of stay was 6 (IQR:4 – 9) days. Additionally, most of the patients had a history of prior hospital admission (56.8%, 92/162) with no ICU admission (98.8% 160/162), antibiotic use (65.4%, 106/162), invasive procedure (71.6%, 116/162), no nasogastric tube (NGT) use (67.3%, 109/162), and the majority were discharged alive from the ICU (80.9%, 131/160), Table 1.

**Table 1:** Socio-demographic and clinical characteristics of study participants

Patient characteristics	Frequency (n)	Percent (%)
Age		
Mean (SD)	44.21 ± 17.42	
Gender		
Male	104	64.2
Female	58	35.8
Primary reason for admission		
RT conditions	36	22.2
CV conditions	27	16.7
Cancer	20	12.3
Brain infection	13	8.0
GIT infections	8	4.9
Kidney disorder	11	6.8
Fractures	11	6.8
Kidney disease	12	7.4
Liver disorder	7	4.3
Post-surgery complications	5	3.1
Anaemia	4	2.5
Burns	4	2.5
Soft tissue injuries	3	1.9
Arthritis	1	0.6
Underlying health condition (n = 106)		
Hypertension	52	49.1
Diabetes	34	32.1
HIV	14	13.2
Cancer	6	5.7
Anemia	5	4.7
Cardiac failure	2	1.9
Hepatitis B	2	1.9
Hypothyroidism	1	0.9
Referral status		
Referral from other facilities	49	30.2
Non-referral	113	69.8
LOS (Median (IQR) days	6(IQR:4 – 9)	
Short (≤ 5 days)	76	46.9
Median (6—10 days)	55	34
Long (> 10 days)	31	19.1
History of prior antibiotic use within last 30 days (n = 106)		
Amoxiclav	39	24.1
Amoxycillin	13	8
Ceftriaxone	25	15.4
Levofloxacin	10	6.2
OABS	19	11.7
Prior ICU admission in the last 30 days		
Yes	2	1.2
No	160	98.8
Invasive procedure done		
Yes	116	71.6
No	46	28.4
Prior hospitalization in the last 30 days		
Yes	92	56.8
No	70	43.2
With NGT		
Yes	53	32.7
No	109	67.3
Samples collected		
Ascitic tap	1	0.6,
Blood	45	27.8
Wound swab	27	16.7
Trachea aspirate	19	11.7
Throat swab	1	0.6
Stool	1	0.6
Urine	67	41.4
Sputum	1	0.6

*NGT* nasogastric tube, *OABS* other antibiotics, *IQR* interquartile range, *ICU* intensive care unit, *SD* standard devistion, *GIT* gastrointestinal tract, *LOS* length of stay, *HIV* human immunodeficiency virus, *RT* respiratory tract conditions, *CV* cardiovascular condition

### Gram-negative bacteria spectrum and infections

**Fig. 1:**
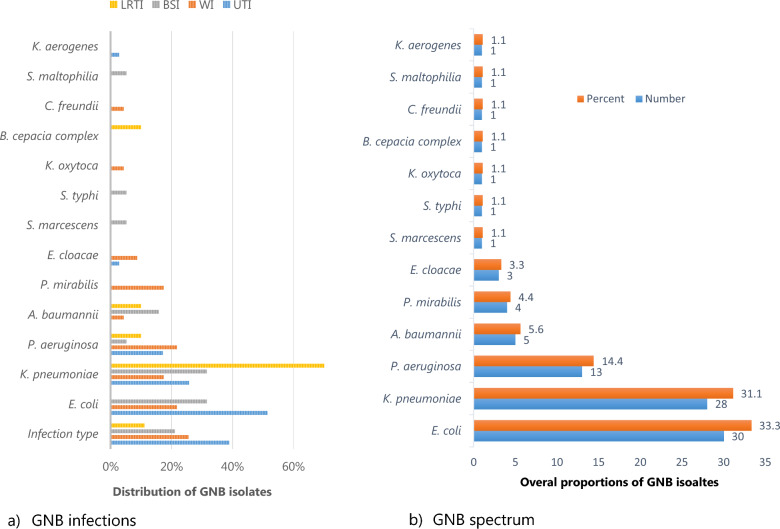
GNB spectrum and infections. *GIT* gastrointestinal tract infections, *URTI* upper respiratory tract infections, *LRTI* lower respiratory tract infections, *BSI* bloodstream infections, *WI* wound infections, *UTI* urinary tract infections, *GNB Gram-negative bacteria*

Ninety patients (90/162; 55.6%) had Gram-negative bacteria infections, with urinary tract infections (UTI) (35/90, 39%) being predominant, Fig. 1a. *Escherichia coli* (18/35, 51%) and *Klebsiella pneumoniae* (7/10, 70%) were the leading cause of urinary tract and lower respiratory tract infections, respectively. Other infections caused by *K. pneumonia* and *E. coli* were ascites, gastrointestinal and upper respiratory tract infections, and accounted for 3% of all GNB infections. Overall, we recovered thirteen [13] Gram-negative bacteria (GNB) types, whereby *Escherichia coli* (30/90, 33.3%) and *Klebsiella pneumoniae* (28/90, 31.1%) were the most common isolates, Fig. 1b.

### Factors associated with Gram -negative bacterial infections

Patients referred from other hospitals were more than two times at risk of GNB infection when compared to those admitted directly to our study site (cOR = 2.23, 95% CI 1.13–4.70, p = 0.025). Participants with a history of antibiotic use were four times more likely to have GNB infection(aOR = 4.23, 95% CI 1.77–10.11, p =0.001). Those on nasogastric tube were three times at risk of harbouring GNB (aOR = 3.04, 95% CI 1.26–7.32, p = 0.013). Patients with respiratory tract were five times more likely to have GNB infection (aOR = 5.3, 95% CI 1.67–16.75, p= 0.005), while those with cardiovascular conditions were six times at risk (aOR = 5.7, 95% CI 1.25–25.81, p = 0.024), Table 2.

**Table 2:** Factors associated with Gram-negative bacterial infection among study participants

	GNB infection		cOR (95%CI)	p-value	aOR (95%CI)	p-value
	Yes n(%)	No n(%)				
Age						
< = 5 years	4(4.4)	2(2.8)	0.80(0.11–6.10)	0.231	–	–
6–17 years	3(3.3)	3(4.2)	1.60(0.23–11.27)	0.637	–	–
18–29 years	14(15.6)	4(5.6)	0.46(0.10–2.21)	0.33	–	–
30–41 years	19(21.1)	10(13.9)	0.84(0.22–3.26)	0.804		
42–53 years	27(30.0)	23(31.9)	1.36(0.39–4.75)	0.627		
54–65 years	15(16.7)	25(34.7)	2.67(0.74–9.67)	0.312		
> = 65 years	8(8.9)	5(6.9)	Ref.			
Gender						
Male	53(58.9)	51(70.8)	0.59(0.31–1.14)	0.139	0.51(0.24–1.09)	0.082
Female	37(41.1)	21(29.2)	Ref.		Ref.	
Pathology						
Respiratory tract conditions	16(17.8)	20(27.8)	2.5(0.96–6.50)	0.06*	5.3(1.67–16.75)	0.005**
CVD	19(21.1)	8(11.1)	4.5(1.15–17.65)	0.031*	5.69(1.25–25.81)	0.024*
Cancer	11(12.2)	9(12.5)	1.64(0.53–5.02)	0.389	2.63(0.7–9.92)	0.153
Brain infection	4(4.4)	9(12.5)	1.2(0.25–5.87)	0.822	1.15(0.34–3.91)	0.819
GIT infection	5(5.6)	3(4.2)	3.5(0.85–14.34)	0.082	1.61(0.26–10.09)	0.613
Kidney disorder	4(4.4)	7(9.7)	1.14(0.28–4.68)	0.853	3.9(0.82–18.66)	0.088
Fractures	7(7.8)	4(5.6)	1.11(0.56–3.22)	0.755	1.14(0.23–5.6)	0.871
Others	24(26.7)	12(16.7)	Ref.		Ref.	
Referral status						
Yes	34(37.8)	15(20.8)	2.31(1.13–4.70)	0.025*	2.15(0.87–5.33)	0.099
No	56(62.2)	57(79.2)	Ref.		Ref.	
LOS						
Short (≤ 5 days)	39(43.3)	37(51.4)	Ref			
Median (6—10 days)	31(34.4)	24(33.3)	1.73(0.73–4.086)	0.215	-	–
Long (> 10 days)	20(22.2)	11(15.3)	1.41(0.57–3.49)	0.461	-	–
Prior antibiotics use within last 30 days						
Yes	69(76.7)	37(51.4)	3.11(1.59–6.09)	0.001**	4.23(1.77–10.11)	0.001**
No	21(23.3)	35(48.6)	Ref.		Ref.	
Invasive procedure						
Yes	67(74.4)	49(68.1)	1.37(0.69–2.71)	0.386	–	–
No	23(25.6)	23(31.9)	Ref.			
Prior hospitalization in the last 30 days						
Yes	56(62.2)	36(50)	1.65(0.88–3.09)	0.151	–	–
No	34(37.8)	36(50)	Ref.			
Using NGT						
Yes	39(43.3)	14(19.4)	3.16(1.55–6.49)	0.001**	3.04(1.26–7.32)	0.013*
No	51(56.7)	58(80.6)	Ref.		Ref.	

The bolded values were the statistical significant values at *P* < 0.001, indicating a strong evidence against the null hypothesis

*ICU* Intensive care unit, *cOR* crudes Odds Ratio, *aOR* adjusted Odds Ratio, *LOS* length of stay, *RT* respiratory tract, *CV* cardiovascular, *GIT* gastrointestinal tract, *GNB*, Gram-negative bacteria, *Ref* reference, *CI* confidence interval, *NGT* nasogastric tube, statistically significant at p < 0.05

**statistically significant at p < 0.001

### Antimicrobial susceptibility profiles of GNB isolates

Generally, Enterobacteriaceae isolates showed resistance to third-generation cephalosporins (3GCs), ranging from 50 to 100%, Table 3. Further*, Escherichia coli*, *Klebsiella pneumoniae, Enterobacter cloacae*, and *Proteus mirabilis* exhibited tigecycline resistance (13 to 100%). *Klebsiella pneumoniae* (46% to 54%) and *Escherichia coli* (10 to 27%) dominated carbapenem-resistant *Enterobacteriaceae* (CRE), and the highest carbapenem resistance (CR) (60% to 100%) was among non-fermenting GNB, including *Acinetobacter baumannii* and *Pseudomonas aeruginosa*. *Escherichia coli* and *Klebsiella pneumoniae* were also resistant to colistin (17 to 46%). Colistin resistance in *Acinetobacter baumannii* and *P. aeruginosa* and *S. maltophilia*, ranging from 60 to 92%, was recorded, but *A. baumannii* remained susceptible to tigecycline, Table 3.

**Table 3:** Antimicrobial susceptibility profiles of isolates

ABS class	ABS	P	*E. coli *[30] (%)	*K. pneumoniae *[28] (%)	*P. aeruginosa *[13] (%)	*A. baumannii *[5] (%)	*P. mirabilis *[4] (%)	*E. cloacae *[3] (%)
AMINO	AMK	R	13	54	31	80	0	0
		S	87	46	69	20	100	100
	GEN	R	23	68	69	100	50	0
		S	77	32	31	0	50	100
Penicillin	AMP	R	73	100	100	100	75	67
		S	27	0	0	0	25	33
	AMC	R	30	64	100	100	0	100
		S	70	36	0	0	100	0
	PIP	R	17	54	54	100	0	0
		S	83	46	46	0	100	100
1GC	CFZ	R	77	96	100	100	50	100
		S	23	4	0	0	50	0
2GC/BLI	C/T	R	57	75	54	100	50	100
		S	43	25	0	0	50	0
3GC	CTX	R	73	93	100	100	50	100
		S	27	7	0	0	50	0
	CAZ	R	70	89	69	100	50	100
		S	30	11	31	0	50	0
	CRO	R	73	89	85	100	50	100
		S	27	11	15	0	50	0
4GC	FEP	R	63	93	69	100	50	100
		S	37	7	31	0	50	0
Sulfonamides	SXT	R	77	89	92	100	75	100
		S	23	11	8	0	25	0
Nitrofurans	NIT	R	27	68	92	100	100	67
		S	73	32	8	05	0	33
Quinolones	CIP	R	83	82	69	80	50	100
		S	17	18	31	20	50	0
	LVX	R	70	79	62	80	50	67
		S	30	21	38	20	50	33
Glycylcyclines	TGC	R	13	21	85	0	100	67
		S	87	79	15	100	0	33
Carbapenems	ETP	R	27	54	85	100	0	0
		S	73	46	15	0	100	100
	IMP	R	10	50	77	100	0	0
		S	90	50	23	0	100	100
	MEM	R	27	46	69	100	0	0
		S	73	54	31	0	100	100
Polymyxins	CST	R	17	46	92	60	100	0
		S	83	54	8	40	0	100

*AMP* ampicillin, *AMC* amoxicillin-clavulanic acid, *PIP* piperacillin, *AMK* amikacin, *GEN* gentamicin, CFZ-cefazolin, *C/T* ceftolozane-tazobactam, *CTX* cefotaxime, *CAZ* ceftazidime, *CRO* ceftriaxone, *FEP* cefepime, *SXT* trimethoprime-sulfamethazole, *NIT* nitrofurantoin, *CIP* ciprofloxacin, *LVX* levofloxacin, *TGC* tigecycline, *ETP* ertapenem, *IMP* imipinem, *MEM* meropenem, *CST* colistin, *S* susceptible, *R* resistant, *P phenotype, ABS antibiotics, AMINO aminoglycosides, 1GC first-generation cephalosporin, 2GC second-generation cephalosporin, 3GC third-generation cephalosporin, 4GC fourth-generation cephalosporin*

The antimicrobial susceptibility profile of isolates less than three, including Stenotrophomonas maltophilia, Salmonella typhi, Klebsiella oxytoca, Klebsiella aerogenes, Burkholderia cepacia complex, and Citrobacter freundii, was not presented.

### Multidrug resistance

Ninety two percent (92%) of the GNB isolates in this study were multidrug-resistant (MDR), with *Escherichia coli* (27/30, 90%)*, Klebsiella pneumoniae* (25/28, 89.3%)*,* and *Pseudomonas aeruginosa* (13/13, 100%) as the most frequent isolates, Table 4. *Salmonella typhi* was non-MDR.

**Table 4:** MDR among isolates

Bacterial isolates	Number of isolates (N)	Non-MDR, *n (%)*	MDR,* n (%)*
*Escherichia coli*	30	3 (10)	27 (90.0)
*Klebsiella pneumoniae*	28	3 (10.7)	25 (89.3)
*Pseudomonas aeruginosa*	13	0 (0.0)	13 (100.0)
*Acinetobacter baumannii*	5	0 (0.0)	5 (100.0)
*Proteus mirabilis*	4	0 (0.0)	4 (100.0)
*Enterobacter cloacae*	3	0 (0.0)	3 (100.0)
OGNB	7	1 (14.3)	6 (85.7)
Frequency	90	7(7.8)	83 (92.2)

*OGNB* other GNB, including, *Stenotrophomonas maltophilia* (1), *Klebsiella oxytoca* (1), *Klebsiella aerogenes* (1), *Citrobacter freundii* (1), *Burkholderia cepacia complex* (1), and *Salmonella typhi* (1); *MDR* multidrug-resistant

## Discussion

In this study, 56% of patients admitted to Intensive care units (ICUs) had Gram-negative bacterial infections, a prevalence higher than reported in Tanzania [18] Nigeria [19], Nepal [20], Ethiopia [21] and Mexico [22] but lower than documented in the city of Sakaka in Turkey and Saudi Arabi [23]. Frequently ICU-admitted patients require medical interventions involving invasive procedures and mechanical devices, and they have induced immunosuppression and comorbidities that increase their risk for nosocomial infections (NI) [24]. Up to 30% of patients admitted to ICU in developed countries acquire at least one NI, whereas, in low and medium-income countries (LMICs), the frequency is at least 2–3 times higher [25], and mortality is reportedly higher (33.6%) than in high-income countries (< 20%) [26].

Urinary tract infections (UTI, 39%) and wound infections (26%) were the most common condition in ICU-admitted patients in the current study, and overall, *Escherichia coli* (33.3%) and *Klebsiella pneumoniae* (31.1%) were the most common isolates. The distribution of infections and their leading etiologies differ widely in the published literature. Sadar et al. found pneumonia (61.4%) as the most common infection in ICU-admitted patients from United States hospitals (2018 − 2020), with *Pseudomonas aeruginosa* (23.5%), *Escherichia coli* (18.8%)*, *and *Klebsiella pneumoniae* (14.4%) as the predominant isolates. Elsewhere in an adult ICU of University Hospital Center in Marrakesh-Morocco, El mekes and others reported pneumonia (39%), bacteremia (29%), and catheter-related blood-stream infections (17%) as the most common infections [27]. Siwakoti and others reported *Acinetobacter* species (41%) as the leading cause of GNB infections, followed by *Klebsiella pneumoniae* (28%) and *Pseudomonas spp* (21%), in a Nepalian ICU [20]. Agaba and others found *Klebsiella pneumoniae* (30%) and *Acinetobacter* species (22%) as the most predominant GNB infections in Ugandan ICUs [28], whereas, in Mexican ICUs, *P. aeruginosa, K. pneumoniae, E. coli,* and *A. baumannii* were the most prevalent pathogens [22]. In a scoping review of infections and antimicrobial resistance in ICUs in LMICs, Saharman and colleagues found that *Acinetobacter baumannii*, *Pseudomonas aeruginosa*, and *Klebsiella pneumoniae* were the predominant isolates [1]. *Klebsiella pneumoniae* was the predominant isolate reported from January 2021 to March 2022 in the ICU of the Southern Medical University of Shunde Hospital, Foshan City, Guangdong Province, China [29].

In ICUs, the epidemiology of GNB infections may vary based on adherence to infection prevention and control policies and the patient's demographic and clinical characteristics. According to the Tripartite Antimicrobial Resistance Country Self-assessment Survey or TrACSS), the system established to monitor country's progress towards the implementation of the AMR global action plan, 11% of 162 countries did not have an IPC programme or an operational plan in 2021–2022, 54% had either unimplemented national IPC programmes/operational plans or implementation was taking place in selected health facilities, and only 34% of countries were having an IPC programme implemented nationwide. Less than 25% of countries in the World Health Organization (WHO) African region had an IPC programme, national and facility-level IPC guidelines, IPC education and training, and IPC monitoring, evaluation and feedback [30].

Here, patients with a history of antibiotic use were four times more likely to have GNB infections when compared to those without. Antibiotic overuse can increase the risk of more severe, prolonged and recurrent infections due to antimicrobial-resistant pathogens [31] and the antimicrobials associated-negative health effects may vary, ranging from direct drug toxicity to dysbiosis and immune cell dysfunction to idiosyncratic drug reactions [32]. Our study finding underscores the importance of balancing patients’ antibiotics-associated harm with the need for prompt and appropriate therapy [32] and emphasizing strict adherence to antimicrobial stewardship policies. Further, patients using nasogastric tubes (NGT) were three times at risk of harbouring a GNB infection in this study. This finding is consistent with that of Despotovic and others in a Serbian ICU [33] that NGT can predispose patients to pathogenic GNB colonization within 48 to 72 h [34] or even within the first day of the tube insertion [35], suggesting pre-insertion contamination. These tubes are reportedly associated with aspiration pneumonia in artificially ventilated patients [29, 36] and increase mortality [33].

In this study, participants with respiratory tract and cardiovascular conditions were five and six times, respectively, more likely to have a GNB infection. Bacterial infections play a crucial role in the pathogenesis of cardiovascular diseases (CVD). For instance, *Chlamydia pneumoniae, Porphyromonas gingivalis,* and *Helicobacter pylori* infections increase the risk of CVD [37]. Simonsen and others reported an increased risk of cardiovascular disease in bacterial infections among individuals with type 1 diabetes [38]. Further, patients with pulmonary comorbidity are especially prone to GNB [39], mainly due to impaired innate immunity that predispose them to bacterial colonization and infection of the respiratory tract [40]. Generally, patients with chronic diseases, such as coronary and respiratory diseases, have prolonged mechanical ventilation time, length of stay, and suppressed immunity making the patient more vulnerable to infection [41].

In the current study, *Enterobacteriaceae *isolates showed third-generation cephalosporins (3GCs) resistance, ranging from 50 to 100%. Third-generation cephalosporins-resistant *Enterobacteriaceae* (3GCRE), including *Escherichia coli, Klebsiella pneumoniae,* and *Enterobacter cloacae*, were recently reported in Kenya among severely ill COVID-19 patients [13], from ‘high-touch’ sites in multiple hospital departments [42], and in communities and hospitals settings [43]. These bugs appear among the top World Health Organization (WHO) global priority pathogens (GPP), along with carbapenem-resistant- *Enterobacteriaceae*, -*Pseudomonas,* and *-Acinetobacter baumannii*, categorized as critical due to drug resistance and the need to discovery and development of new antimicrobial agents [44], In *Enterobacterales*, 3GC resistance is predominantly due to the production of extended-spectrum β-lactamases (ESBL) [45] and 3GCRE-strains pose higher disease burden than carbapenem-resistant ones [46]. Further, ESBL-producing *Enterobacteriaceae* often exhibit multidrug resistance and increase in their prevalence favour over-prescription of carbapenems, the drugs of ‘last-resort’ for treatment of multidrug-resistant (MDR) GNB infections, with consequent exacerbation of carbapenem resistance in our study setting.

Here, *Klebsiella pneumoniae* and *Escherichia coli* dominated carbapenem-resistant *Enterobacteriaceae* (CRE), whereas among non-fermenting GNB, *Acinetobacter baumannii* and *Pseudomonas aeruginosa* were the leading carbapenem-resistant isolates*. *Additionally, 90% of *Escherichia coli* (27/30)*, *89.3% of *Klebsiella pneumoniae *(25/28)*, *and 100% of *Pseudomonas aeruginosa* (13/13) were MDR. CR genes can rapidly spread in clinical isolates via horizontal gene transfer involving plasmids, transposons, and integrons, and these elements often harbour multiple antibiotic-resistance genes [47]. Multidrug-resistant and carbapenem-resistant pathogens present a critical global health challenge [46] and are currently a growing clinical problem in Kenya [12, 43, 47, 48]. They cause community- and hospital-acquired pneumonia and complicated urinary tract infections, bloodstream infections, and complicated intra-abdominal infections. With limited antibiotic options for infections caused by CR pathogens, polymyxins are the mainstay therapy. However, reports on colistin-resistant clinical isolates are increasing globally, suggesting diminishing treatment options for CR-GNB infections and a high risk of difficult-to-treat (DTT) pathogens [20, 49]. We observed colistin resistance, ranging from 60 to 100%, among *A. baumannii* and *P. aeruginosa *isolates. Widespread antibiotic use in agriculture and pisciculture is among the leading drivers of drug resistance [50]. In a study by Kariuki and others on antibiotic use by poultry farmers in Kiambu County, Kenya, 13% of farmers used colistin in poultry feeds [51]. The public health implications of colistin-resistant pathogens in our setting remain critical because newer treatment options for CR bacterial infections, including ceftazidime/avibactam and meropenem/vaborbactam, are costly and largely unavailable.

## Conclusion

We report a high prevalence of MDR-GNB infections, predominated by urinary tract infections, in ICU, whereby patients with a history of antibiotic use, using the nasogastric tube, and having respiratory tract and cardiovascular conditions were at increased risk. To improve the management of ICU-admitted patients, continuous education, training, monitoring, evaluation and feedback on infection prevention and control (IPCs) are warranted in our study setting.

## Study limitation

This was a single hospital-based study, and bacteria isolates molecular characteristics were not elucidated due to limited resources. However, the data presented show a high burden of MDR-GNG infections in a country where most healthcare facilities lack microbiology laboratories or laboratories inadequately equipped and poorly supplied, with most antibiotic prescriptions not guided by antibiograms.

## Data Availability

This study’s primary data will be available on request.

## Abbreviations

CLSI: Clinical and Laboratory Standard Institute
AMR: Antimcrobial resistance
GNB: Gram negative bacteria
MDR-GNB: Multidrug resistant Gram negative bacteria
ICU: Intensive care unit
UTI: Urinary tract infections
CR: Carbapenem Resistant
WBC: White blood cells
NGT: Nasogastric tube
CRE: Carbapenem-resistant *Enterobacteriaceae*
CRPA: Carbapenem-resistant *Pseudomonas aeruginosa*
CRAB: Carbapenem-resistant *Acinetobacter baumannii*
